# Sudden hearing loss subsequent to diarrhea: what is the missing link?

**DOI:** 10.1186/2008-2231-22-15

**Published:** 2014-01-08

**Authors:** Gholamali Jafari, Seyed Mohammadreza Hosseini, Shahin Akhondzadeh

**Affiliations:** 1 Forensic Medicine Organization, Tehran, Iran; 2 Psychiatric Research Center, Roozbeh Hospital, Tehran University of Medical Sciences, Tehran, Iran

**Keywords:** Diarrhea, Hearing loss, Metronidazole, Ototoxicity

## Abstract

Sudden sensorineural hearing loss (SSNHL) is a debilitating condition with an incidence of nearly 20 per 100,000 in populations. Metronidazole-induced ototoxicity is an extremely rare etiology of SSNHL. In this report, we describe a young female with bilateral SSNHL due to oral use of metronidazole. A 23 years old female presented to the emergency department with acute bilateral hearing loss. We found out that her hearing loss had started 4 days after initiation of metronidazole which was administered for treatment of diarrhea. This case report shows that physicians should be aware of the uncommon side effects while prescribing metronidazole to patients in order to manage the possible adverse events on time.

## Background

Sudden sensorineural hearing loss (SSNHL) is a subset of sudden hearing loss (SHL) which affects nearly to 20 per 100,000 in populations [1]. Due to its debilitating nature and sudden course, patients often immediately seek medical care. If SSNHL is diagnosed and managed on time, chance of hearing recovery as well as patient quality of life will be increased. For diagnosis of SSNHL, hearing impairment should have rapid onset, occurring over a 72-hour period, and a reduction in hearing of ≥30 decibels (dB) and at least 3 successive audiometric frequencies should be present in the audiometric test. Based on the latest clinical practice guidelines, computerized tomography should not be ordered in the initial evaluation of these patients [2]. Although SSNHL has a very long list of etiologies, the etiology remains unknown in nearly 90% of cases despite thorough evaluation [3]. Thus, due to uncertain etiology in most cases, treatment of SSNHL is mainly empiric and usually consists of corticosteroids, [4] antivirals, antibiotics, diuretics and mineral supplements [4,5]. Also, if a potential drug is suspected to cause SSNHL, it must be stopped promptly.

## Case presentation

A 23 years old female presented to the emergency department with acute bilateral hearing loss. Her problem had first started 3 days ago by hearing a non-pulsatile sound and tinnitus in both ears. Her hearing had been progressively decreasing and worsened markedly within the next 3 days. She denied any ear pain and drainage, headache, vertigo or oral lesion and did not have fever and chills. There was no history of antecedent fluctuating hearing loss, head trauma, acoustic trauma, allergic rhinitis, sinusitis or recurrent urinary tract infection. She reported acute watery diarrhea in the past week. She had history of a rhinoplasty surgery 9 months previously. Initial history revealed no regular medication use. The patient did not smoke and did not use alcohol or illicit substances.

The patient was alert and nervous. She could not hear verbal communication clearly, thus the physician used handwritten notes to communicate with the patient and vice versa. Her vitals were as follows: body temperature was 37.4°C, blood pressure was 110/65 mm Hg, heart rate was 100 beats per minute, and her respiratory rate was 18 breaths per minute. General physical examination was normal except for mild tenderness of both feet to light touch. The otoscopic examination of both ear canals and tympanic membranes were normal. There was no tragus tenderness. She had negative whisper sign on both sides. A complete neurologic examination was done and was normal.

The patient was admitted to the ear, nose and throat ward with impression of sudden bilateral sensorineural hearing loss ematologic lab tests and biochemistry were done and all parameters were in normal range. She was negative for rheumatoid factor and had normal erythrocyte sedimentation rate (ESR) and C-reactive protein (CRP) levels. Audiometry of both ears confirmed SNHL (30-dB hearing loss at 3 consecutive frequencies).

The initial empiric treatment was started immediately and included: bilateral intratympanic dexamethasone, oral prednisolone and oral acyclovir. There was no significant improvement by day 4 of admission. Due to increased foot pain and tenderness, the patient was admitted to the rheumatology ward for further investigation. Serologic studies for known autoimmune diseases were normal and viral markers were negative. Methotrexate, pentoxifylline and folic acid were added to the aforementioned treatment regimen. Ten days after treatment initiation, gradual improvement of hearing was reported by the patient. Also, serial audiogram at 10th day showed significant improvement especially in the right ear at low frequencies.

We continued to search for the etiology of SSNHL in the patient by interviewing her and reviewing the history several times on different occasions. Serendipitously, we found out that her hearing loss had started 4 days after initiation of metronidazole which was administered for treatment of diarrhea.

## Discussion

In this report we present an extremely rare etiology of bilateral SSNHL. There are only three metronidazole-associated SSNHL reports in the literature [6]. Metronidazole is a 5-nitromidazole antibiotic which is widely used against anaerobic bacteria and protozoal infections. In general, metronidazole has a relatively safe drug profile and is well tolerated but there are several reports that high doses or prolonged administration can on rare occasions cause serious adverse events. Different mechanisms have been suggested for metronidazole-induced neurotoxicity including inhibition of protein synthesis, vasogenic and cytotoxic edema and mitochondrial dysfunction. To date, the exact mechanism still remains unknown. Interestingly it has been reported that metronidazole can augment gentamycin-induced ototoxicity [7].

The patient described in this report presented to the Emergency Department with bilateral SHL. Audiogram confirmed the diagnosis of SSNHL. Despite complete physical examination and laboratory studies, the underlying cause remained unknown until we interviewed the patient several times and discovered metronidazole use. Early treatment and supportive care eventually reversed the SNHL although not completely. After 2 weeks of hospitalization, she was eventually discharged with oral folic acid, pentoxifyllin and calcium supplements.

Recently a systematic review found no association between dose or duration of metronidazole use and rare side effects [8]. In our patient, metronidazole was administered 750 mg orally 3 times daily for 10 days for treatment of symptomatic Blastocystis Hominis infection. She noted that as the diarrhea and GI symptoms resolved on the 4th day of the treatment, she discontinued the drug. Although this is the recommended dose by Center for Disease Control for treatment of Blastocystis infection and the patient did not complete treatment for the prescribed duration, she experienced bilateral SSNHL 96-hours after the treatment was commenced.

## Conclusion

Taken all together, this case suggests that physicians should be aware of the uncommon side effects while prescribing metronidazole to patients in order to manage the possible adverse events on time.

## Consent

Written informed consent was obtained from the patient.

## Abbreviations

CRP: C-reactive protein; ESR: Erythrocyte sedimentation rate; SSNHL: Sudden sensorineural hearing loss.

## Competing interest

No competing interest exists for any of the authors associated with the manuscript.

## Authors’ contributions

SMRH and SA drafted the manuscript. AJ and SMRH: contributed to acquisition of data and, interpreted the data. All authors read and approved the final manuscript.
